# An Online Survey of the Perceptions of Clinical and Non-Clinical Professionals on Healthcare for Non-Communicable Diseases and COVID-19 Measures During the Pandemic in Malaysia

**DOI:** 10.3389/ijph.2023.1605861

**Published:** 2023-05-25

**Authors:** Sugitha Sureshkumar, Feisul Mustapha, Haironi Yusoff, Kibachio Joseph Mwangi, Kailing Marcus, Bogomil Kohlbrenner, David Issom, Mohamed-Rida Benissa, Sigiriya Aebischer-Perone, Nirit Braha, Egidio Candela, Kumar Gaurav Chhabra, B. R. Desikachari, Arianna Dondi, Marina Etchebehere, Gladwell Gathecha, Andre Pascal Kengne, Eduardo Missoni, Benjamin Palafox, Sanghamitra Pati, Priyanka Paul Madhu, Nasheeta Peer, Jennifer Quint, Reza Tabrizi, Michel Oris, David Beran, Dina Balabanova, Jean-Francois Etter

**Affiliations:** ^1^ Institute of Global Health, University of Geneva, Geneva, Switzerland; ^2^ Disease Control Division, Ministry of Health (Malaysia), Putrajaya, Malaysia; ^3^ Department of Medicine and Public Health, Universiti Malaysia Sarawak, Kota Samarahan, Malaysia; ^4^ Department of Non-Communicable Diseases, Ministry of Health (Kenya), Nairobi, Kenya; ^5^ University Hospitals of Geneva, Geneva, Switzerland; ^6^ NHS England, London, United Kingdom; ^7^ IRCCS Azienda Ospedaliero, Universitaria di Bologna, Bologna, Italy; ^8^ NIMS Dental College and Hospital, NIMS University, Jaipur, India; ^9^ Medical Council of India, New Delhi, India; ^10^ Faculty of Health Sciences (FICSAE), Hospital Israelita Albert Einstein, São Paulo, Brazil; ^11^ Non-Communicable Diseases Research Unit, South African Medical Research Council, Cape Town, South Africa; ^12^ CERGAS SDA Bocconi, Milano, Italy; ^13^ London School of Hygiene and Tropical Medicine, University of London, London, United Kingdom; ^14^ Regional Medical Research Center (ICMR), Bhubaneswar, India; ^15^ Sharad Pawar Dental College and Hospital (SPDC), Wardha, India; ^16^ Imperial College London, London, United Kingdom; ^17^ Noncommunicable Diseases Research Centre, Fasa University of Medical Sciences, Fasa, Iran; ^18^ Faculty of Medicine, University of Geneva, Geneva, Switzerland

**Keywords:** COVID-19, pandemics, non-communicable diseases, Malaysia, healthcare quality

## Abstract

**Objectives:** This study assesses the opinions of health professionals in Malaysia on the disruption of non-communicable disease (NCD) services during the COVID-19 pandemic from March 2020 to January 2022.

**Methods:** We conducted a cross-sectional online survey with 191 non-clinical public health workers and clinical health service workers in Malaysia from November 2021 to January 2022. Participants were recruited by the Malaysian Ministry of Health using major networks including key experts and practitioners. Secondary respondents were subsequently enrolled through snowballing.

**Results:** The most notable issues raised by the survey participants relate to NCD service disruption, the redirection of NCD care resources, and NCD care being overburdened post-pandemic. Respondents also reported accounts of resilience and prompt reaction from the healthcare system, as well as calls for innovation.

**Conclusion:** Most respondents perceived that the challenges arising from COVID-19 were mostly managed well by the healthcare system, which was able to provide the necessary services to NCD patients during this health emergency. However, the study identifies gaps in the health system response and preparedness capacity, and highlights solutions for strengthening NCD services.

## Introduction

The COVID-19 pandemic brought unprecedented challenges for the resilience of healthcare systems globally [1]. It affected healthcare systems by diverting technical, economic and human resources from other vital services. This includes care for non-communicable diseases (NCDs), which encompass detection, screening, treatment and palliative care for the management of chronic conditions.

In the specific context of a low- and medium-income Asian country, the Malaysian health system delivers a comprehensive range of services through public and private providers, addressing NCDs [2]. Despite the probability of dying between ages 30 and 70 years from one of the four main NCDs, namely, cancer, diabetes, cardiovascular diseases and chronic respiratory diseases, declining from 19.3% in 2005 to 17.2% in 2016, NCDs continue to account for 71% of the burden of disease in Malaysia [2].

Malaysia has a strong health system financed mainly through general revenue and taxation, and further through out-of-pocket payments and private health insurance for those seeking private care [6]. However, 23% rural population are disassociated to health facilities, and have a higher prevalence of NCDs [3, 4].

The National Strategic Plan for Non-Communicable Diseases (NSP-NCD) 2016–2025, published by the Ministry of Health (MoH) of Malaysia in 2016, formulates a roadmap to combat NCDs with input from the National Health and Morbidity Survey (NHMS) [7]. Since the first NHMS in 1986, successive surveys have found an astounding rise in the prevalence of NCDs [7], leading the MoH to recognize NCDs as one of the ‘biggest challenges to the current healthcare system in Malaysia’ [7]. The MoH developed the NSP-NCD in response, based also on the World Health Organization’s (WHO) Global Action Plan for the Prevention and Control of NCDs 2013–2020 [7].

The emergence of COVID-19 impacted NCD patients, 20% of whom were predisposed to severe illness should they become infected with the virus [8]. From the first confirmed patient with COVID-19 in Malaysia in January 2020 through to October 2022, the country witnessed 4.8 million confirmed cases and over 36,000 deaths [9]. Persons living with cardiovascular diseases (CVD), diabetes, cancers and those with CVD risk factors such as hypertension and obesity were at increased risk of hospital admission, severe disease and death from COVID-19, and they were also at risk of added complications from poorly controlled chronic disease through poor follow up [10].

Surveys of patient perceptions have cast light on diminished NCD services and standards in different country contexts during the pandemic [11, 12], but few studies have endeavoured to document the perceptions of healthcare personnel regarding the performance and readiness of the health system during the pandemic. By surveying health professionals we can understand their views on the preparedness capacities and the impact of the pandemic on NCD services in Malaysia. This, in turn, may help efforts to strengthen NCD services, resources and management following the pandemic or in preparation for future crises, by providing new insights and ensuring the acceptability of adjustments. Moreover, the Malaysian experience may inform countries with a similar level of development.

Therefore, the objective of this study was to assess and compare the opinions of health professionals in Malaysia (public health officials, health policymakers and healthcare workers) on NCD care and on the health system preparedness and response in the context of the COVID-19 pandemic.

## Methods

### Participants and Sampling

MoH Malaysia provided access to study participants, but the study design, implementation, and data analysis and interpretation were independent from the MoH. We conducted a cross-sectional online survey among health personnel in Malaysia working at all levels from primary to tertiary care and in health offices, from November 2021 to January 2022 (Table 1). We chose an online survey for time and resource efficiency, as well as participant accessibility. Two participant groups were included: non-clinical public health and health policy officials (herein “non-clinical workers”) and clinical health service workers (herein “clinical workers” [i.e., doctors, nurses, midwives]). The following inclusion criteria were applied: being employed in Malaysia in one of the two categories above, in a governmental or in a non-governmental organization.

**TABLE 1:** Description of Sample (Geneva, Switzerland. 2022).

Federal territory/State	Population in state [41, 42] (% of country total 32,655,400)	Density of doctors; nurses (per 1,000 population) [42]	Proportion of respondents by province (% of total 191)
Kuala Lumpur	1,746,600 (5%)	3.0; 8.5	7
Johor	379,400 (12%)	1.2; 2.7	8
Kedah	2,194,100 (7%)	1.3; 2.7	5
Kelantan	1,928,800 (6%)	1.1; 2.9	3
Labuan	100,100 (0.3%)	0.8; 2.4	3
Malacca	937,500 (3.6%)	1.9; 4.2	4
Negeri Sembilan	1,129,100 (3%)	1.9; 3.3	4
Pahang	1,684,600 (5%)	1.3; 3.0	3
Penang	1,774,400 (5%)	1.8; 4.4	5
Perak	2,508,900 (7%)	1.4; 3.1	5
Perlis	255,400 (0.7%)	2.1; 3.8	0.5
Putrajaya	116,100 (0.4%)	52.6; 35.0	5
Sabah	3,833,000 (12%)	0.8; 2.1	9
Sarawak	2,822,200 (9%)	1.3; 2.5	7
Selangor	6,555,400 (20%)	1.5; 2.7	7
Terengganu	1,275,100 (4%)	1.4; 2.7	1
Not disclosed	—		23

Total Respondents *n* = 191.

Major networks including key experts and practitioners with broad vision and knowledge of the field were identified collaboratively with the government and the researchers. The emphasis was on networks involved in policy development and having the expertise, long term knowledge of the health system and contributing to policy development. We emailed survey invitations to 87 individuals within networks. Using the snowballing sampling technique, respondents forwarded the survey link to further professionals to invite them to participate. In total 191 health personnel participated in the survey: 117 (61%) clinical health workers and 74 (39%) non-clinical health workers. The study respondents had a combined average work experience of 9.6 years in health services, with the majority of participants having worked between five and 10 years in health services.

We used the Limesurvey platform for data collection. Both clinical and non-clinical respondents answered over 75% of the survey questions. Participants provided informed consent online and we did not collect any identifying information. The intended sample size was 200, which would have allowed us to obtain a 95% confidence interval of ±7% for variables whose frequency was 50%, and to detect a 20% difference between groups for dichotomic variables whose frequency was 50%, with a power of 80% and a *p*-value of 0.05.

### Measurements

A literature analysis, expert group discussions, and a phone survey of four Malaysian healthcare personnel (this preliminary survey covered their perception of NCD care during the pandemic) enabled us to identify five study themes: 1. Health system preparedness (policy readiness and implementation capacity), 2. Emergency preparedness (processes in place to counter adverse public health scenarios to NCD services), 3. Allocative efficiencies (distribution and redistribution of human, economic and technical resources), 4. Governmental/policy perceptions (ideas and opinions of officials within the health governing structure), 5. Innovation (possible future initiatives to address adverse public health scenarios). An additional theme was also conceptualized to ascertain NCD status in the country and better understand the pre-COVID situation. The questionnaire was then pre-tested qualitatively on the same four individuals for intelligibility, exhaustiveness and accuracy, and improved iteratively during two rounds.

The online questionnaire included 49 multiple choice questions covering the status of NCD care during the COVID-19 pandemic (23 questions), plus the health system preparedness and responses to the pandemic, suggestions for changes to NCD services and the utility of information technology (26 questions) (Supplementary Material S1). We used two distinct questionnaires, one for non-clinical workers that excluded questions pertaining to patient interactions, and one for clinical workers that excluded questions on policy-making decisions. The questions and response options were prioritized in consultation with experts from 12 countries.

### Data Entry and Analysis

Data downloaded from LimeSurvey were kept within a secure server at the University of Geneva. STATA (release 17.0, StataCorp LLC, College Station, TX) was used to calculate means for continuous variables, percentages and frequencies to describe categorical variables, and Chi-square tests to compare proportions. In Tables 2, 3, we report percentages calculated using valid answers as the denominator.

**TABLE 2:** Summary of survey findings—NCD status in Malaysia and COVID-19 Health System Response in Malaysia, online survey (Geneva, Switzerland. 2022).

	Survey question	Response	Clinical workers %	Non-clinical workers %	*p*-value	Chi^2^
NCD status in Malaysia						
1	Rate NCD care in your country	Good + excellent	55	36	<0.001	32.84
2	Are you aware of an integrated HIV/TB/Family Planning and NCD plan in Malaysia?	No	26	52	<0.001	17.55
3	Would you agree/disagree that the traditional delineation of Infectious and NCD care should be eliminated to provide the holistic care that seems imperative in countering damaging outcomes during infectious epidemics?	Agree	57	100	<0.001	13.10
4	Has children’s care in your institution been compromised in any way?	Yes	46	48	0.652	0.86
5	What priority was NCD care accorded in your country prior to the COVID-19 pandemic?	Minimal	—	46	—	—
6	Did this priority change considering the heightened risk of severe disease among patients with pre-existing conditions?	Yes	—	40	—	—
COVID-19 health system response in Malaysia						
7	What was the amount of beds created for the surge response?	Large	76	44	<0.001	25.41
8	Were patients who otherwise would receive a cubicle/isolated space in a department/ward, now being placed in shared space/ward beds?	Yes	54	—	—	—
9	Were multidisciplinary teams created intentionally for working in COVID-19 wards? (i.e., redistribution of specializations to internal medicine, respiratory medicine, ICU for the care of COVID-19 patients)	Yes	63	81	0.02	7.34
10	Were health professionals briefed on infection protection control and parallel public health measures?	Yes	100	100	—	—
11	Was the dedicated personnel often rotated so as not to tire them?	Yes	70	70	0.98	0.00
12	Did the dedicated personnel have any training focusing on COVID-19?	Yes	73	55	0.003	11.24
13	Did you have the feeling that there was some confusion about how to treat a COVID-19 patient: i.e., frequent changes in drug indications and dosages, protocols?	Yes	100	83	0.002	9.49
14	If there was a redistribution of doctors and nurses unfamiliar with high intensity internal medicine care, were they placed on COVID-19 patient wards regularly (long enough to learn protocols and manage well)?	Yes	65	50	0.003	15.93
15	Do you worry that NCD patients who have not been followed up adequately due to the crisis (and not clinically afflicted by COVID-19) will have worsened outcomes when it comes to their NCD pathology due to this compromised care?	Yes	65	100	0.003	11.64

Note: Frequency in percentage; *p*-value from chi^2^; missing *p*-value and chi^2^ due to no comparison group or expected value <5.

Total respondents *n* = 191.

**TABLE 3:** Summary of survey findings—Malaysian Health System Preparedness and Adaptation online survey (Geneva, Switzerland. 2022).

	Survey question	Response	Clinical workers %	Non-clinical workers %	*p*-value	Chi^2^
Malaysian health system preparedness and adaptation						
1	Did you feel the political decision makers in your country were aware of the risk to frontline workers?	Quite a bit + very	—	68	—	—
2	Did your healthcare workers receive adequate personal protection equipment dependant on their workplace setting?	No	3	32	<0.001	34.99
3	If you were on the frontline (in emergency departments, on respiratory, or ICU wards) taking care of Sars-CoV-2 positive patients, did you receive adequate personal protection equipment (PPE)?	Completely	83	—	—	—
4	How would you rate the severity of NCD service disruption (including elective surgery, follow-up clinics, routine care) during the pandemic?	Occasional + very often	54	57	0.5	2.39
5	Was the provision of individual protection devices adequate or inadequate?	Adequate	54	—	—	—
6	Were there contingency plans to shield the system in case of an external disruption like COVID-19?	No	30	28	<0.001	38.07
7	Do/did you anticipate an over burden on the NCD services when routine care eventually resumed?	Yes	59	36	<0.001	27.9
8	Do/did you have plans for gradual/spaced out resumption of routine care?	Yes	79	16	<0.001	60.23
9	Do you plan to evaluate the medical impact of this pandemic on NCDs?	Yes	—	40	—	—
10	Do you plan to evaluate the social and economic burden of this pandemic on NCDs management?	Yes	—	42	—	—
11	Is there a plan in place to screen the potential mental health impact of the pandemic?	Yes	—	43	—	—
12	As those with NCDs are the most vulnerable subgroups of patients, do you plan to target increased prevention, health education programmes towards them?	Yes	—	54	—	—
13	As those with NCDs are the most vulnerable subgroups of patients, do you plan to target increased outreach initiatives (using volunteers, increased personnel, etc.) and address accessibility (using telemedicine, mobile pharmacies, etc.)?	Yes	—	61	—	—
14	Would you agree/disagree with the need to ensure that whole populations should have increased health promotion, prevention schemes addressed to them to decrease vulnerabilities and predispositions to NCDs, with the goal of optimizing prognoses in the time of infectious epidemics?	Agree	57	100	<0.001	28.29
15	Would you consider using digital health applications for medical information sharing, especially for patients in multidisciplinary care settings?	Yes	75	83	0.484	1.45
16	Would you agree that digital health (eHealth or mHealth) can be integrated into your healthcare infrastructure to better care for your most vulnerable patients in crises such as this?	Disagree + strongly disagree	68	32	<0.001	15.77
17	To better maintain the normal flow of follow-ups and care of your country’s NCD patients, how likely are you to depend on telehealth, digital health, mobile health applications?	Very much	80	48	<0.001	29.12
18	Taking into account health literacy, access to mobile devices, and financial aspects, would digital health be an option for NCD patients to experience more autonomy over their care?	Yes	80	86	0.28	2.55
19	Did your country’s health system cope with the redistribution of resources (both human and economic) at the cost of continuous care of NCD patients?	Yes	—	70	—	—
20	Did you reorient national/regional guidelines and protocols to concentrate services in a setting suited to high-volume, high-acuity care available 24 h per day?	Yes	41	5	<0.001	36.16
21	Was redirecting chronic disease management to focus on maintaining supply chains for COVID-19 an option?	Yes	52	19	<0.001	20.64
22	If redirection of chronic care was an option, how successful was it in terms of delivery of care?	Met standards	25	68	<0.001	30.22
23	If redirection of chronic care was an option, how successful was it in terms of output?	Met + above standards	68	58	0.17	1.85

Note: Frequency in percentage; *p*-value from chi^2^; missing *p*-value and chi^2^ due to no comparison group or expected value <5.

Total respondents *n* = 191

### Ethics

The study protocol was approved by the Jawatankuasa Etika & Penyelidikan Perubatan (Medical Research and Ethics Committee) of the MoH Malaysia (protocol number NMRR-20-3156-57734 IIR) on 08/04/21, and all study procedures upheld the 1964 Helsinki Declaration. The study proceeded without deviation from the proposal.

## Results

We received survey responses from 191 participants (Table 1). Most (61%) respondents were clinical workers and the remainder were non-clinical (39%, split into 23% public health officials and 16% health policy workers). Study participants were invited from institutions across all levels of function and from all states in Malaysia. Most states were well represented and the geographical distribution of participants was roughly proportionate to the distribution of the general population (Table 1).

### NCD Status in Malaysia

NCD services in Malaysia were considered “good” or “excellent” by 55% of clinical workers and by 36% of non-clinical workers, whereas the remainder felt it was either satisfactory, or not good. Almost half (46%) of non-clinical respondents reported that Malaysia considered NCDs as a “minimal priority” before the COVID-19 pandemic, and 40% felt that this priority level changed during the pandemic because of the heightened risk of severe illness from COVID-19 among patients with pre-existing conditions (Table 2).

With regards to models of care, 57% of clinical workers and all (100%) non-clinical workers agreed that the tradition of care stratified for infectious diseases and NCDs should be eliminated to provide the holistic care that seems imperative to counter damaging outcomes during epidemics.

### COVID-19 Response in Malaysia

Looking at capacity, 76% of clinical workers and almost half (44%) of non-clinical workers stated that a “large” amount of hospital beds were created during the pandemic (Table 2). All those who responded in both groups stated (100%) that infection control and parallel public health measures were widely known by health professionals (Table 2).

Across both participant groups, most (72%) answered that multidisciplinary teams of clinicians were created to work in COVID-19 wards, and 70% answered that the dedicated personnel rotated to manage workloads. About half (55%) said that health service workers were deployed to COVID-19 patient wards regularly, i.e., long enough to learn protocols and manage cases adequately.

All (100%) clinical workers said there was some confusion about COVID-19 treatments (i.e., frequent changes in protocols) and 73% stated that personnel received specific training on COVID care. This compares with most (83%) non-clinical workers who believed there was some confusion about how to treat a COVID-19 patient and half (55%) who said specific training was provided.

Two-thirds (65%) of clinical workers and all (100%) non-clinical workers expressed the concern that NCD patients who were not followed up adequately due to the crisis would have worsened NCD outcomes.

### Health System Preparedness and Adaptation During the COVID-19 Pandemic

#### Health System Disruption

Almost all respondents rated the disruption to chronic disease management as at least “occasional” (95% clinical workers; 100% non-clinical workers), and 42% overall rated this disruption as “severe” (Table 3). Between 28% and 30% of all respondents reported that no contingency plans were in place before the pandemic to shield the health system from external disruptions. Over half (59%) of clinical workers anticipated that NCD services would be overburdened when routine care eventually resumed and 36% of non-clinical workers believed the same. Most (79%) clinical workers attested to the existence of plans for the gradual resumption of routine care, but few non-clinical workers said the same (16%).

One-quarter (25%) of clinical workers reported that accepted standards were met for the delivery of NCD care in cases of redirected chronic patient care, whereas 68% of non-clinical workers felt such standards were met. Regarding the outputs of the implemented redirection of NCD services, similar proportions (68% and 58%, respectively) of clinical and non-clinical workers stated that these outputs met or exceeded accepted standards (Table 3).

Most (83%) clinical workers said that personal protection equipment (PPE) was distributed adequately to frontline workers. Most (70%) non-clinical workers perceived that Malaysia’s health system coped with the redistribution of resources (both human and economic) but at the cost of continuous care of NCD patients.

#### Health Promotion and Prevention of NCDs

With regard to future action, 57% of clinical workers and all (100%) non-clinical workers believed that the whole population should have access to increased health promotion/prevention schemes to decrease vulnerabilities and predispositions to NCDs.

A majority (61%) of non-clinical workers stated that they would strengthen outreach initiatives to NCD patients, including increased community health worker activity and health education programmes, while half (54%) planned to target increased prevention and health education programmes for NCD patients.

#### Digital Health

Most (75% clinical and 83% non-clinical) participants reported that they would consider using digital health applications for medical information-sharing in multidisciplinary care settings, with most (80% clinical, 86% non-clinical) viewing these innovations as useful in helping patients to acquire more autonomy. While 32% of clinicians “agreed” or “strongly agreed” that electronic health platforms/mobile health applications (eHealth/mHealth) should be integrated into the healthcare infrastructure to better care for the most vulnerable patients, twice as many (68%) non-clinical workers stated the same (Table 3).

Most (80%) clinical workers said that they were “very likely” to depend on telehealth, digital health and mhealth applications to provide continuous care and follow-up for NCD patients, and 48% of non-clinical workers responded comparably.

### Summary of Findings

Using a cross-sectional design, this study presents the responses of clinical and non-clinical workers across Malaysia to evaluate perceptions of the impact of COVID-19 on NCD services. As might be expected, clinical health workers—who were primarily frontline physicians and nurses—saw the impact of the pandemic through their own point of contact with the health system (i.e., clinics, hospitals). Public health officials and staff, on the other hand, expressed a broader view across services (clinics, hospitals and outreach services in their respective jurisdictions).

There is a clear difference between the responses of clinical and non-clinical health workers on particular issues. A larger proportion of clinical health workers (50%) rated the NCD care in Malaysia as “good + excellent” during the pandemic compared to non-clinical health workers (32%). Over half (57%) of clinical workers agreed to the elimination of the traditional delineation of infectious and NCD care compared to all non-clinical workers. And half (50%) of clinical workers anticipated that NCD services would be overburdened once routine services resume compared with just over one-quarter (28%) of non-clinical workers.

However, a similar proportion of clinical (35%) and non-clinical (33%) workers expressed concern that NCD patients who had not been followed up during the pandemic will have worse outcomes post-pandemic. Meanwhile, a similar—relatively low—proportion of both clinical (37%) and non-clinical (28%) health workers agreed that the pandemic had severely disrupted NCD health services. Regarding health promotion, fewer (41%) clinical health workers agreed to increase health promotion and prevention schemes compared to non-clinical health workers (64%). Looking at future models of care, 61% of clinical workers versus 15% of non-clinical health workers agreed that they are likely to depend on telehealth, digital health and mhealth applications to better maintain a normal flow of NCD follow-up care post-pandemic. Similarly, 67% of clinical workers versus 26% of non-clinical health workers agreed that mobile devices would give patients greater autonomy in their healthcare, regardless of literacy and financial status.

## Discussion

This study provides insight on the impact of COVID-19 on NCD services in Malaysia and how the healthcare system responded, by documenting the perceptions of frontline clinicians as well as public health and policy officers. NCDs are a barrier to development, and most LMICs see the value in averting its detrimental effects on their health systems’ technical and operational capacities, as well as progress in meeting development goals [13]. A crisis setting, inherently inhibits the implementation strength of priority interventions, including health promotion and prevention, access to essential drugs, and innovation. Crisis on a global scale impedes international cooperation, effectiveness of governance structures, and progress towards the sustainable development goals [13–15].

### Comparison of Clinical and Non-Clinical Respondents

Clinical and non-clinical workers in our sample differed in their responses about many issues. These pertain to the quality of NCD services, the response of the healthcare system, the redirection of chronic care, plans for the gradual resumption of care, the prevention of NCDs and digital health. The variation in opinions between clinicians and non-clinicians are likely explained by differences in their access to information, work experience and professional networks, and in dissimilarities in their roles, tasks, motivations and expectations, as has been previously reported [26, 27]. This highlights the importance of canvassing direct feedback from all stakeholders to ensure future cooperation, and demonstrates that changes implemented at the policy level have an impact at the grassroots of service delivery.

### The Health System Response

Significant disruptions to NCD services as a consequence of COVID-19 have been reported in many countries, including those with greater resources than Malaysia [16]. According to WHO, almost 77% of all countries worldwide experienced health service disruption during the pandemic, including disruption to the provision of NCD care [17]. Mitigation measures, including movement restrictions and other public health measures, had a negative impact on access to services in many countries, to the detriment of health outcomes [17]. To varying degrees, the study participants expressed concern regarding NCD service disruption during the pandemic, the redirection of NCD care and the potential for services to be overburdened post-pandemic. However, the responses to our survey also reveal the resilience and effective response of the Malaysian healthcare system, with substantial proportions of respondents indicating that they witnessed adequate planning and resourcing to counter the adverse consequences for NCD care. Many LMICs report that guidance from WHO and other humanitarian/international organizations helped develop contextually appropriate adaptations to navigate the disruption caused by COVID-19. These included facility-level administrative and engineering controls, changes in clinical practices, including routine management, and a shift to remote consultations and increased scope of community healthcare [18]. A pivot from routine chronic and acute care was aimed to optimize the utilization of resources, although the effectiveness on maintaining the existing standards of NCD care in many settings is unknown due to incomplete or ongoing data collection [19].

Despite these tremendous efforts, a notable shortfall reported by respondents, included inadequate guidance for redirecting healthcare from chronic to acute services, and confusion regarding protocols for COVID-19 case management. The latter can be explained by the frequent changes in clinical guidelines as the pandemic progressed and as rapid scientific advances were made [20]. Studies in other settings show that the redirection of care was increasingly challenging in other countries too, due to a lack of comprehensive packages that include NCD guidelines essential medicines and innovations, and this remains an important point for improvements [21–24].

Both participant groups in the present study reported concerns about worsened health outcomes due to compromised care, with inadequate follow-up for NCDs and interruptions of both elective procedures and continuous care. Furthermore, the literature shows that COVID-19 and NCDs have a reciprocal effect on each other [25]. The drivers of these syndemic effects must be addressed so that NCD patients avoid worsened outcomes and so that care for NCD patients during crises is included in national response frameworks [25].

### Allocation and Redistribution of Resources

The two participant groups had differing perceptions on the number of hospital beds created during the pandemic. In particular, the answers of non-clinical workers suggest that they felt that more could have been done in this area, even though Malaysia fared well compared to many countries in Asia that were not able to accommodate all patients needing care [28]. In January 2021, there were 33,270 beds allocated to COVID-19 patients in Malaysia across hospitals, quarantine centres and low-risk treatment centres, out of 44,117 existing beds countrywide. Some experts estimated that this bed capacity was more than sufficient [29, 30].

Clinical workers responded that PPE was distributed adequately, while most non-clinical workers expressed the view that resources (both human and economic) were redistributed at the cost of the continuous care of NCD patients. Other studies have found that inequities and capacity issues made it difficult to provide quality care to the Malaysian population during the COVID-19 pandemic [30].

There were also differences among respondents in awareness of the training provided, and MoH Malaysia have internal documents to suggest that there were varying methods used to share the trainings to different professional groups. Previous research shows that uncertainty during a novel pandemic yields inadequate understanding, ambiguous or unreliable information, and conflicting alternatives within a crisis setting [31]. Understanding this uncertainty and communicating about ever-changing protocols and guidelines can enable shared decision-making and better operational crisis management, while also reducing distress [32]. This is a consideration for future pandemic preparedness.

Finally, unlike clinical workers, very few non-clinical workers knew of plans for the gradual resumption of routine care after the crisis, even though MoH plans existed, heavily relying on living guidelines regularly published by the WHO [33]. MoH planned to revert back clinical settings and work flows that were dedicated for COVID-19 patients in stages, had in place special programmes, i.e., implementation of mass surgery, to catch up on delayed elective procedures, and mobilized resources to manage long COVID. COVID-19 assessment centres (CAC) were to be closed down in stages with redeployed healthcare personnel returning back to their original duty stations. Information dissemination is key during crisis situations. More fluid and transparent knowledge-sharing systems will need to be discussed to avert a mismatch in practice and service [32].

### NCD Prevention

Study participants underlined the need for better health promotion and prevention to decrease the NCD burden in the population—although far more non-clinical than clinical respondents expressed this view, possibly due to the concerted public health efforts and interests of non-clinical workers in this area. The current Malaysian NSP-NCD highlights the provision of preventive healthcare services and the promotion of healthy lifestyles, with emphasis given to private and NGO involvement alongside government initiatives [7]. In particular, the Plan suggests that programmes promoting self-management strategies for NCDs might improve health outcomes, including in rural communities, which is an important point given that 23% of the population lives in rural areas [3, 34]. To increase the value of public health planning initiatives, clinical health workers should have greater capacity building and awareness of health promotion and prevention management rather than purely clinical intervention of NCDs.

Looking at models of care, the survey questioned respondents on the traditional delineation of infectious and NCD care. Integrated NCD and infectious disease care might be preferable, including concurrent surveillance, in order to address the increased susceptibility to communicable disease in individuals with NCDs [35]. Non-clinical respondents in Malaysia agreed with this approach, while clinical workers less so, perhaps because a focus on specialization is particularly important to clinical practitioners.

### New Information Technology

Participants widely recognized the benefits of new information technologies and telemedicine to improve health awareness, education, knowledge management and information-sharing, and the management of patient follow-up. It has been noted elsewhere that knowledge-sharing improves policy implementation and facilitates the use of health-related scientific evidence [36]. Most participants also said that NCD patients would experience more autonomy in their care with digital innovation, with research showing that digital technologies can help manage chronic illnesses during disasters or crises, when patients need more autonomy due to restricted access to care and greater isolation [37]. Digital health tools are currently, and will be in the future, an integral part of NCD management during pandemics. Their application should be supported in all countries in preparation for the next crisis [38–40]. To revert back to routine care post pandemic, plans were put in place by MoH to allow the continuation of various digital health applications such as virtual consultations. The MySejahtera App, used for COVID-19 purposes, was repurposed to a more general public health app, with various other modalities, to include general health screening, mental health screening and interventions, smoking cessation, national immunisation programme functions, as well as others.

### Limitations

The questionnaire was not standardized and the questions were validated qualitatively by a small test group only, with no validation statistics prior to the main survey. The questions were screened through the entire study group yet some imperfections lie here. Further, our results depend on self-reports, which reflect the knowledge, information, experience and sincerity of study participants, and therefore may be affected by response bias. The response rate could not be calculated as responses were anonymous and the number of requests sent to secondary participants through snowballing from the initial 87 notifications is unknown. Also, missing answers limited the quality of some questionnaire items, some questions may be leading, the and yes/no answers may not reflect the whole range of participants’ perceptions.

Given the above, the results of our study may not be representative of all healthcare professionals in Malaysia. However, the distribution of our sample across states roughly matches the geographic distribution of the general population. We recognize that the sample size is relatively small and that the opinions of patients and other stakeholders have not been investigated in the present study. Finally, the absence of a control group (i.e., a pre-pandemic survey) limits our ability to interpret the data.

Limitations aside, the assets of this study include its originality and timeliness, the nationwide sample, and the identification of gaps and strengths of the Malaysian healthcare system from the point of view of health workers.

### Conclusion

To varying degrees, the respondents felt that NCD care was affected during the COVID-19 pandemic in Malaysia, but that the healthcare system was mostly able to cope and provide the required care despite unprecedented challenges. Respondents also pointed at some gaps in the health system response and preparedness capacity, however.

Our survey of healthcare workers can inform future planning for NCD strategies in Malaysia, within a crisis context and beyond. It offers insight on the views of health personnel on potential approaches to strengthen NCD services that can guide policy and practice. Ultimately, strengthened NCD care will improve the outcomes for patients of non-communicable and communicable diseases, and this may prove essential during future crises.
