# Supplementary material for: An Online Survey of the Perceptions of Clinical and Non-Clinical Professionals on Healthcare for Non-Communicable Diseases and COVID-19 Measures During the Pandemic in Malaysia
Source: Int J Public Health. 2023 May 25;68:1605861. doi: 10.3389/ijph.2023.1605861 (PMC10247991; doi:10.3389/ijph.2023.1605861)
Supplement: Supplementary file 1 [file DataSheet1.PDF]

Supplement 1. COVID-19-ELEPHANT Questionnaire (Malaysia)

Questions and offered responses		Groups Posed to	
		CW	NCW
1	Rate NCD care in your country.<br>*not good, good, satisfactory, good, excellent*	X	X
2	Do you have examples of unique challenges for NCD patients during the COVID 19 pandemic?<br>*yes, no, unsure*	X	X
3	Does your country have an integrated HIV/TB/Family Planning and NCD plan that you are aware of?<br>*yes, no, unsure*	X	X
4	Would you agree/disagree that the traditional delineation of Infectious and Non-Communicable Disease care should be eliminated to provide the holistic care that seems imperative in countering damaging outcomes during infectious epidemics?<br>*agree, disagree*	X	X
5	Has children's care in your institution been compromised in any way?<br>*yes, no, unsure*	X	X
6	What priority was NCD care accorded in your country prior to the COVID 19 pandemic?<br>*minimal, mediocre, high*		X
7	Did this priority change considering the heightened risk of severe disease among patients with pre-existing conditions?<br>*yes, no, unsure*		X
8	Choose following: no COVID beds; not many COVID beds, sufficient beds, large amount of COVID beds were created<br>*not many, sufficient, large, unsure*	X	X
9	Did you, yourself, work with COVID-19 patients?<br>*yes, no, unsure*	X	
10	What was the level of care given to patients? [Intensive care]<br>*some, most, none*	X	
11	What was the level of care given to patients? [Sub-intensive (high dependency)]<br>*some, most, none*	X	
12	What was the level of care given to patients? [Acute]<br>*some, most, none*	X	
13	What was the level of care given to patients? [Post-acute]<br>*some, most, none*	X	
14	Were patients who otherwise would receive a cubicle/isolated space in a department/ward, now being placed in shared space/ward beds?<br>*yes, no, unsure*	X	
15	Were there COVID 19-dedicated hospitals in your city/region/country?<br>*yes, no, unsure*		X

16	Were multidisciplinary teams created intentionally for working in COVID wards? (ie; redistribution of specializations to internal medicine, respiratory medicine, ICU for the care of COVID 19 patients)<br>*yes, no, unsure*	X	X
17	Were health professionals briefed on infection protection control and parallel public health measures?<br>*yes, no, unsure*	X	X
18	Was the dedicated personnel often rotated so as not to tire them?<br>*yes, no, unsure*	X	X
19	Did the dedicated personnel have any training focusing on COVID?<br>*yes, no, unsure*	X	X
20	Did you have the feeling that there was some confusion about how to treat a COVID patient: e.g. frequent changes in drug indications and dosages, protocols?<br>*yes, no, unsure*	X	X
21	If there was a redistribution of doctors and nurses unfamiliar with high intensity internal medicine care, were they placed on COVID-19 patient wards regularly (long enough to learn protocols and manage well) or irregularly (not allowing them to obtain required skill sets for adequate care)?<br>*yes, no, unsure*	X	X
22	How important was the presence of an adequate support by Palliative Care Specialists as resource to deal with end-stage disease patients (and their family)?<br>*not important, important, very important*	X	X
23	Do you worry that NCD patients who have not been followed up adequately due to the crisis (and not clinically afflicted by COVID 19) will have worsened outcomes when it comes to their NCD pathology due to this compromised care?<br>*yes, no, unsure*	X	X
24	Were there contingency plans to shield the system in case of an external disruption like COVID 19?<br>*yes, no, unsure*	X	X
25	Did your healthcare workers receive adequate personal protection equipment dependant on their workplace setting?<br>*yes, no, unsure*	X	X
26	Was redirecting chronic disease management to focus on maintaining supply chains for COVID 19 an option?<br>*yes, no, unsure*		X
27	Did you feel the political decision makers in your country were aware of the risk to frontline workers?<br>*not at all, a little, quite, very*		X
28	If you were on the frontline (in emergency departments, on respiratory, or ICU wards) taking care of Sars-CoV-2 positive patients, did you receive adequate personal protection equipment?<br>*not at all, a little, quite, completely*	X	

29	Was the provision of individual protection devices adequate or inadequate?<br>*adequate, inadequate*	X	
30	Do/did you anticipate an over burden on the NCD services when routine care eventually resumed?<br>*yes, no, unsure*	X	X
31	Do/did you have plans for gradual/spaced out resumption of routine care?<br>*yes, no, unsure*	X	X
32	How would you rate the severity of NCD service disruption (including elective surgery, follow-up clinics, routine care) during the pandemic?<br>*never, occasional, very often, severe*	X	X
33	Do you plan to evaluate the medical impact of this pandemic on NCDs?<br>*yes, no, unsure, not applicable*		X
34	As those with NCDs are the most vulnerable subgroups of patients, do you plan to target increased prevention, health education programmes towards them?<br>*yes, no, unsure*		X
35	As those with NCDs are the most vulnerable subgroups of patients, do you plan to target increased outreach initiatives (using volunteers, increased personnel, etc.) and address accessibility (using telemedicine, mobile pharmacies, etc.)?<br>*yes, no, unsure*		X
36	Do you plan to evaluate the social and economic burden of this pandemic on NCDs management?<br>*yes, no, unsure*		X
37	Is there a plan in place to screen the potential mental health impact of the pandemic?<br>*yes, no, unsure*		X
38	Did your country's health system cope with the redistribution of resources (both human and economic) at the cost of continuous care of NCD patients?<br>*not at all, somewhat, very much*		X
39	Do you have any suggestions to avoid this compromise in care for NCD patients for future crises?<br>*yes, no, unsure*	X	X
40	Would you agree/disagree with the need to ensure that whole populations should have increased health promotion, prevention schemes addressed to them to decrease vulnerabilities and predispositions to NCDs, with the goal of optimizing prognoses in the time of infectious epidemics?<br>*agree, disagree, unsure*	X	X
41	Would you consider using digital health applications for medical information sharing, especially for patients in multidisciplinary care settings?<br>*yes, no, unsure*	X	X

42	Would you agree that digital health (eHealth or mHealth) can be integrated into your healthcare infrastructure to better care for your most vulnerable patients in crises such as this?<br>*neither disagree nor agree, strongly disagree, disagree, agree, strongly agree*	X	X
43	To better maintain the normal flow of follow-ups and care of your country's NCD patients, how likely are you to depend on telehealth, digital health, mobile health applications?<br>*not likely, somewhat likely, very likely*	X	X
44	Taking into account health literacy, access to mobile devices, and financial aspects, would digital health be an option for NCD patients to experience more autonomy over their care?<br>*yes, no, unsure*	X	X
45	At which level (primary to level 4) do you anticipate best use of these?<br>*primary, secondary, tertiary, level 4, mixed*		X
46	Did you reorient national/regional guidelines and protocols to concentrate services in a setting suited to high-volume, high-acuity care available 24 hours per day?<br>*yes, no, unsure*	X	X
47	Was redirecting chronic disease management to focus on maintaining supply chains for COVID 19 an option?<br>*yes, no, unsure*	X	X
48	If redirection of chronic care was an option, how successful was it in terms of delivery of care?<br>*below standards, meets standards, above standards, far above standards*	X	X
49	If redirection of chronic care was an option, how successful was it in terms of output?<br>*below standards, meets standards, above standards, far above standards*	X	X

CW: Clinical worker
NCW: Non-clinical worker
